# Case report: Microsatellite instability determination is not always black and white in Lynch syndrome diagnosis

**DOI:** 10.3389/fonc.2024.1396869

**Published:** 2024-06-18

**Authors:** Julieta E. Rodriguez, Damien Vasseur, Mohamed Amine Bani, Odile Cabaret, Sophie Cotteret, Martine Muleris, Veronica Golbarg, David Malka, Thomas Pudlarz, Olivier Caron, Cristina Smolenschi

**Affiliations:** ^1^ Drug Development Department, Gustave Roussy Cancer Campus, Villejuif, France; ^2^ Medical Biology and Pathology Department, Gustave Roussy Cancer Campus, Villejuif, France; ^3^ Medical Oncology Department, Gustave Roussy Cancer Campus, Villejuif, France; ^4^ Biology and Genetics Department, Centre Eugène Marquis, Rennes, France; ^5^ Department of Genetics, Hôpital Pitié-Salpêtrière, Assistance Publique – Hôpitaux de Paris (AP-HP), Sorbonne Université, Paris, France; ^6^ Gastroenterology and Hepatology Department, Institut Mutualiste Montsouris, Paris, France

**Keywords:** discordant MSI, colorectal cancer, next-generation sequencing, circulating DNA, case report

## Abstract

**Introduction:**

Microsatellite instability (MSI) is a genetic marker that is useful in the detection and treatment of Lynch syndrome (Sd). Although conventional techniques such as immunohistochemistry (IHC) and polymerase chain reaction (PCR) are the standards for MSI detection, the advent of next-generation sequencing (NGS) has offered new possibilities, especially with circulating DNA.

**Case report:**

We present the case of a 26-year-old patient with Lynch Sd and a *BRAF*-mutated metastatic colon cancer. The discordant MSI results between the conventional methods and NGS posed challenges in making treatment decisions. Subsequent NGS analysis revealed a high MSI status, leading to participation in an immunotherapy trial, with remarkable clinical response.

**Conclusion:**

This case emphasizes the importance of comprehensive molecular profiling and strong interdisciplinary collaborations, especially in cases with ambiguous MSI results.

## Introduction

Microsatellite instability (MSI) is the genetic fingerprint of a defective DNA mismatch repair (dMMR) system (1), in which the accumulation of mutations occur throughout the genome and is particularly grouped in repetitive regions of microsatellites (2). It is routinely assessed in solid tumors for the initial detection of Lynch syndrome, for treatment orientation, and for cancer prognosis (3).

Most MSI-high (MSI-H) tumors arise sporadically (4), often associated with hypermethylation of the *MLH1* promoter or a mutation in *BRAF V600E* (specifically in colorectal cancer, CRC) (5), while others result from hereditary cancer predisposition syndromes such as Lynch syndrome [originated from a monoallelic germline mutation in one of the four major mismatch repair (MMR) genes: *MLH1*, *MSH2*, *MSH6*, *PMS2*, or the *EPCAM* gene] (6, 7). The tumor phenotype of MSI-H CRC is characterized by a right-sided colon presentation, poorly differentiated mucinous adenocarcinomas, an early-disease onset, and a high response to immune checkpoint blockers (8).

Currently, the gold standard for dMMR detection is immunohistochemistry (IHC) and polymerase chain reaction (PCR) using tumor tissue samples. However, the evolution and the development of next-generation sequencing (NGS) techniques have offered the opportunity to extend MSI-H determination particularly using circulating cell-free DNA (cfDNA) (3). The latter has gained significant interest as it is a minimally invasive and easily repeatable tool that overcomes the problem of spatial and temporal heterogeneity and allows longitudinal monitoring of the disease through iterative sampling (9). Thus, there has been special interest in demonstrating the concordance between the use of conventional techniques and NGS tools in MSI-H detection (for validation as a detection method) with promising results (10, 11). However, discordant results could arise and therefore pose challenges in making treatment decisions. In this paper, we present a case report as an example of this issue.

## Case report

A 26-year-old man with no personal or familial history of cancer was hospitalized after 1 week of fever and abdominal pain. The computed tomography (CT) scan showed a primary right colonic mass associated with local inflammation and free pelvic effusion, without distant metastasis. In this context, he underwent emergency surgery. Pathological specimen revealed R0 resection, a 7-cm infiltrative mucinous adenocarcinoma of the right colon, and two positive lymph nodes out of 47 (T3N1 stage IIIA). Molecular testing on the primary tumor found *KRAS* wild type, *BRAF V600E* mutation, and normal expression of *MLH1*–*MSH2*–*MSH6*–*PMS2* as assessed by IHC. The result of the PCR analysis for MSI detection was considered uninterpretable due to insufficient tumor cells (<20%).

Postoperative imaging was clear, as well as the tumor markers [i.e., carcinoembryonic antigen (CEA) and carbohydrate antigen 19–9 (CA19–9)]. As recommended by the guidelines, between January and July 2018, the patient received 12 cycles of adjuvant chemotherapy with an intravenous (iv) FOLFOX regimen [oxaliplatin, 85 mg/m^2^ iv; 5-fluoruracil (5-FU), 2,400 mg/m^2^ iv over 46 h; 5-FU, 400 mg/m^2^ bolus; leucovorin (LV), 400 mg/m^2^]. Genetic counseling was considered due to the patient’s young age, and a standard constitutional NGS panel was performed, which included the genes *MLH1*, *MSH2*, *MSH6*, *PMS2*, *EPCAM*, *APC*, *MUTYH POLD1*, and *POLE*, without detection of any deleterious mutations (**Table 1**). A class 3 heterozygous VUS (variant of uncertain significance) of the *MSH2* gene located in exon 13 c.2012A>C/p.Asn671Thr was found, and due to the patient’s young age, the panel recommended upper and lower endoscopic surveillance as a Lynch-like case (every 2 years in France).

**Table 1:** Summary of the patient’s test results performed for treatment decision making.

Panel	Microsatellite status	TMB (Muts/Mb)	Gene Alterations
IHC (MMR)			Normal expression by tumor cells of MLH1- MSH2- MSH6- PMS2
Genetic analyse - TEST MSI - PCR			BAT40 instable NR21 instable NR24 instable BAT25 stable; BAT26 stable; D2S123 stable; D5S346 stable; D17S250 stable; NR22 stable
NGS genetic panel			Class 3 heterozygote variant of MSH2 (exon 13 c.2012A>C / p.Asn671Thr)
Genetic analyse – TEST MSI	MSI-High		ACVR2A; BTBD7 (Not mutated: DIDO1; MRE11; RYR3; SEC31A; SULF2)
Genetic analyse – hypermethylation of MLH1 gene promoter			somatic hypermethylation of MLH1 gene promoter was negative
NGS Foundation Medicine (Tissue)	MSI-High	17.65	BRAF V600E; PIK3CA G118D MLL2 G1281*; FBXW7 L497fs*1; MLL2 E888fs*42; RNF43 R117fs*41; NOTCH1 G1894fs*49
Foundation One Liquid CDx	MSI-High	27	BRAF V600E; PIK3CA G118D ATR I774fs*5; CHEK1 T226fs*14 FBXW7 L497fs*1; RNF43 R117fs*41 and P660fs*88 (1.9%); CD79A R131fs*61; EP300 N1700fs*9 and M1470fs*26; MLL2 G1281* and E888fs*42; NOTCH1 G1894fs*49; PBRM1 I279fs*8; TET2 L615fs*24 and G223fs*28; TGFBR2 K128fs*3

Muts/Mb, mutations per megabase; NGS, next-generation sequencing; IHC, immunohistochemistry; MSI, microsatellite instability; MMR, mismatch repair; PCR, polymerase chain reaction

In December 2018, the patient had resectable liver relapse, and a left hepatectomy was performed. As the relapse was intrahepatic and occurred 6 months after the end of adjuvant chemotherapy, the multidisciplinary team (MDT) decided on an adjuvant systemic treatment with an iv FOLFIRI regimen (irinotecan, 180 mg/m^2^; 5-FU, 2,400 mg/m^2^ iv over 46 h; 5-FU, 400 mg/m^2^ bolus; LV, 400 mg/m^2^) plus intra-arterial hepatic oxaliplatin. The patient received 3 months of chemotherapy from February to May 2019, with good tolerance. He was disease free at the end of the treatment.

After 3 months, in September 2019, a CT scan revealed progressive disease and the appearance of retroperitoneal lymph nodes. As the patient harbored *BRAF V600E* mutation, the MDT decided on targeted therapy with dabrafenib, trametinib, and panitumumab [at the time, we did not have the results of the BEACON trial (12)]. The patient had partial response and a progression-free period of 16 months (between September 2019 and February 2021), but with limited tolerance due to grade 2–3 cutaneous toxicity related to panitumumab.

In January 2021, the disease became progressive again at the retroperitoneal, mediastinal, and left supraclavicular lymph nodes. Despite the fact of a known *BRAF V600E* mutation and the proficient mismatch repair (pMMR) status, we decided to perform a new molecular NGS analysis with the FoundationOne CDx panel, which used both liquid biopsy and the archival specimen (from the hepatic surgery).

The results revealed, on tissue, MSI-H, tumor mutational burden (TMB) of 17.65 mutations per megabase (Muts/Mb), and a *BRAF V600E* mutation, while the liquid biopsy confirmed the MSI-H, TMB-H (27 Muts/Mb), and the *BRAF V600E* mutation, among others (**Table 1**).

**Figure 1:**
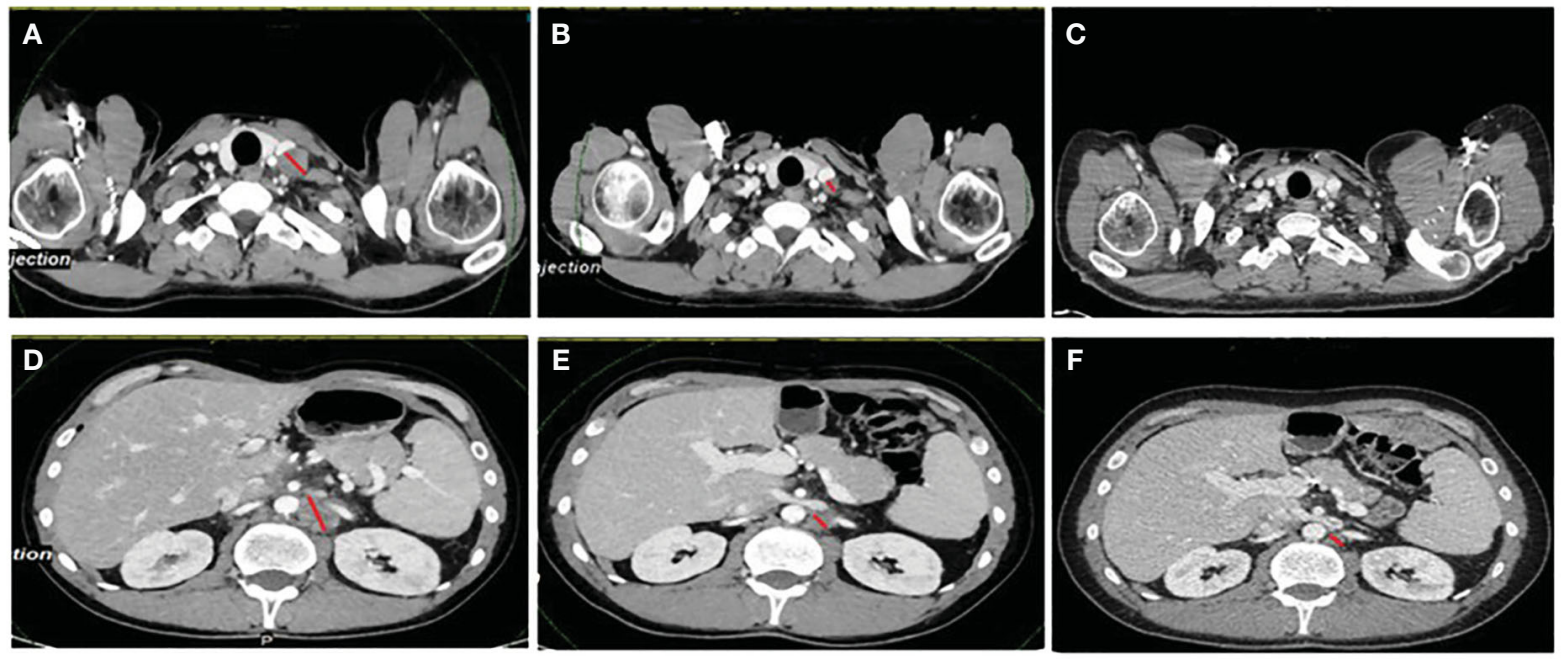
The patient’s CT scans at baseline, at best response, and at the last evaluation. **(A–C)** Evolution of a left supraventricular node. **(D–F)** Retroperitoneal node. The images in **(A)** and **(D)** were taken before starting immunotherapy treatment, while the images in **(B)** and **(E)** are those at the best response (this patient experienced a partial response with a −76% reduction in the target lesions). The images in **(C)** and **(F)** are the last scans of the patient in January 2024, when the decision to stop the treatment was made after 34 months. The red lines denote measurable disease.

**Figure 2:**
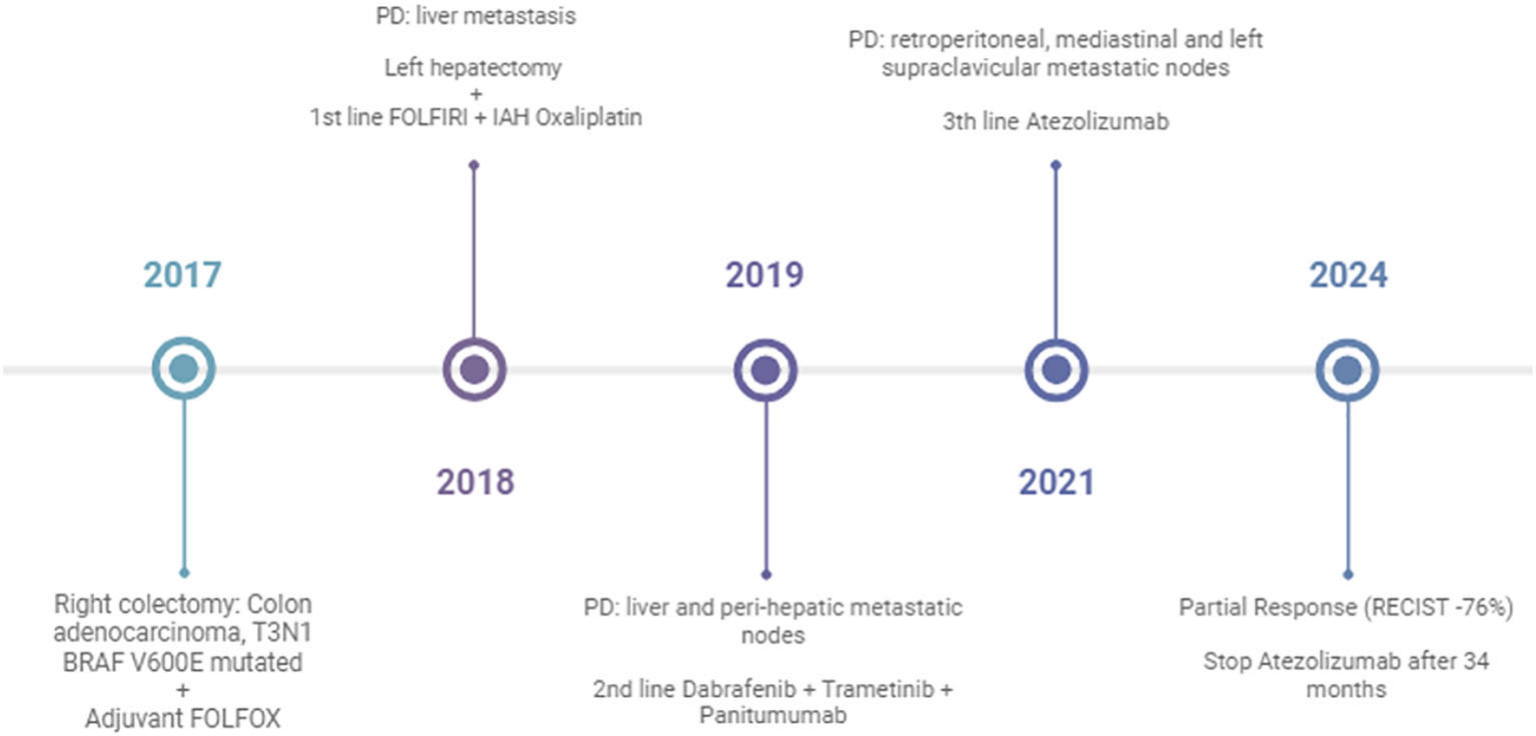
Timeline of the clinical case presentation. PD, progressive disease.

Based on the MSI-H result, the patient was enrolled in a basket clinical trial, in which he underwent treatment for 34 months with second-line atezolizumab with partial response (−76%) and complete metabolic response with excellent treatment tolerance (**Figure 1**). The diagnosis and the treatment process of the patient are displayed in **Figure 2**.

In the face of these discordant results, we performed an MSI analysis using the specimen from the liver metastasis. The analysis revealed an MSI-H phenotype in NGS, but still a microsatellite stable (MSS) in IHC. A second NGS using the Idylla panel confirmed the presence of MSI-H in the liver specimen. The case was discussed with a pathologist and a biologist, and a somatic hypermethylation of *MLH1* was performed, which came back negative. The patient was considered to harbor an MSI-H tumor.

After the MSI-H results were confirmed, we went back on the germline analysis and decided to perform a methylation tolerance-based functional assay (13) for the *MSH2* exon 13 c.2012A>C/p.Asn671Thr. The results confirmed the pathogenicity of the variant, and the case was discussed in genetic MDT. The variant was classified as likely pathogenic (in the national FrOG (14) database), and it was considered that the patient harbored Lynch syndrome. The evaluation performed by the end of January 2024 showed a complete metabolic response, and it was decided to stop immunotherapy. His healthy relatives had not performed any genetic testing at this stage.

## Discussion

We present the case of a 26-year-old male patient with a metastatic colon cancer that presented discordant MSI results between IHC/PCR and NGS. The NGS panel done at progression (liquid biopsy and the most recent tissue) yielded results of MSI-H and TMB-H, offering the possibility to be exposed to immunotherapy with impressive results.

Before DNA sequencing became available, MSI detection mainly relied on IHC for the MMR proteins and PCR evaluation of the five highly conserved loci of the “Bethesda panel” (15). However, given that, on certain occasions, biopsies may not contain a sufficient percentage of tumor cells for correct analysis of the MMR status (as well as other genes of special interest), a significant number of patients may find themselves limited in their therapeutic options (16).

According to the recommendations of the European Society of Medical Oncology (ESMO) and the American Society of Clinical Oncology (ASCO), the MMR status should be assessed in all patients at the time of CRC diagnosis whenever Lynch syndrome is suspected, but also as an initial molecular workup in metastatic disease for its predictive value for the use of immunotherapy [Level of evidence (I,A)] (17, 18). Testing should be carried out using conventional techniques such as MMR-IHC and/or MSI by PCR, which are the primary recommendations. Furthermore, if MMR detection is conducted with an NGS panel, this must show equivalency to the aforementioned techniques (18).

In the context of this case, the outcome of the IHC, indicating a typical expression of the *MSH2* protein despite a missense variant, raises intriguing questions about its functional impact. While Lynch syndrome screening typically relies on IHC and/or MSI tests, with genetic testing reserved for specific cases, further investigations were warranted due to the patient’s young age and the potentially inconclusive MSI test, leading to the accurate identification of the syndrome.

Our patient presented an uninterpretable MSI status from the use of conventional techniques, assessed at the initial diagnosis, probably due to specimen cellularity (<20%) or the mucinous histology. He was treated with standard therapies with short periods of disease control.

Several causes could lead to a false-negative MSI result, as follows: 1) technical issues (MSI testing on tissue fragments <5.5 mm could produce a false-negative MSI result) (19); 2) mucinous histology (20); and 3) some Lynch syndrome (a number of individuals with Lynch syndrome could have tumors with an MSI-L or an MSS phenotype (20–22), leading to false-negative results). Thanks to the high availability of NGS platforms in our center, we were able to discover that the patient had an MSI-H tumor. He was then treated with atezolizumab in the basket trial, which was the only possibility at the time.

In 2020, the Keynote-177 trial presented the results of pembrolizumab treatment in patients with MSI-H, which showed an improvement in progression-free survival and, notably, a response rate of 43.8% compared with standard chemotherapy, then becoming the first-line treatment for this population, even for patients harboring an *BRAF V600E* mutation (23). Approval for pembrolizumab was followed by nivolumab (with or without its combination with ipilimumab) for the same setting, thanks to the results of the phase II trial Checkmate 142 (2022) (24). We would also like to mention that our patient had a *BRAF V600E* mutation, which was considered only until recently to be more a marker for the sporadic MSI cancer. However, of late, the presence of a *BRAF V600E* mutation has also been found in patients with Lynch syndrome; therefore, its presence should not exclude germline testing if clinically indicated (25).

In conclusion, our focus on the MSI status, particularly in younger patients, underscores the critical role it plays in making treatment decisions. When confronted with unclear results, consulting biologists and geneticists become imperative, given the life-changing potential of an accurate diagnosis. If direct determination from the tumor tissue is not feasible, cfDNA is an option in such cases.

## Data availability statement

The data that support the finding of this case are available from the corresponding author upon reasonable request.

## Ethics statement

Written informed consent was obtained from the individual(s) for the publication of any potentially identifiable images or data included in this article.

## Author contributions

JR: Conceptualization, Investigation, Methodology, Visualization, Writing – original draft, Writing – review & editing. DV: Investigation, Writing – review & editing. MB: Formal analysis, Writing – review & editing. OdC: Formal analysis, Writing – review & editing. SC: Formal analysis, Writing – review & editing. MM: Formal analysis, Writing – review & editing. VG: Formal analysis, Writing – review & editing. DM: Formal analysis, Writing – review & editing. TP: Validation, Writing – review & editing. OlC: Formal analysis, Writing – review & editing. CS: Investigation, Supervision, Validation, Writing – review & editing.
